# Correction: Treatment outcomes among snakebite patients in north-west Ethiopia—A retrospective analysis

**DOI:** 10.1371/journal.pntd.0011965

**Published:** 2024-02-16

**Authors:** Inge Steegemans, Kassaye Sisay, Ernest Nshimiyimana, Gashew Gebrewold, Turid Piening, Endale Menberu Tessema, Birhanu Sahelie, Gabriel Alcoba, Fikre Seife Gebretsadik, Dirk Essink, Simon Collin, Emiliano Lucero, Koert Ritmeijer

There are errors in the Funding and Competing Interests statements. The correct statements are:

**Funding:** MSF provided financial support for this study. The snakebite activities were financed under the operational cost of the MSF Ethiopia mission, and MSF provided support for the publication costs. MSF also provided support for this study in the form of salaries for KS, EN, TP, EMT, BS, GA, EL, and KR. MSF had a role in study design, data collection and analysis, decision to publish and preparation of the manuscript. This is indicated by the affiliation of the coauthors, and their specific roles are articulated in the ‘author contributions’ section.

**Competing Interests:** The authors have read the journal’s policy and have the following competing interests to declare: MSF provided support for this study in the form of salaries for KS, EN, TP, EMT, BS, GA, EL, and KR. This does not alter our adherence to *PLOS Neglected Tropical Diseases* policies on sharing data and materials. There are no patents, products in development or marketed products associated with this research to declare.
