# Supplementary material for: Treatment outcomes among snakebite patients in north-west Ethiopia—A retrospective analysis
Source: PLoS Negl Trop Dis. 2022 Feb 9;16(2):e0010148. doi: 10.1371/journal.pntd.0010148 (PMC8863263; doi:10.1371/journal.pntd.0010148)
Supplement: S1 Table — (PDF) [file pntd.0010148.s001.pdf]

Supplement 1

Antivenom products used by Médecins Sans Frontières during the research period.

**Brand name**	**Company**	**Country of production**	**Raised against venom of species**	**Period of use by Médecins Sans Frontières**
Fav-Afrique	Sanofi-Pasteur	France	*Bitis arietans, B. gabonica, Dendroaspis jamesoni, D. polylepis, D. viridis, Echis leucogaster, E. ocellatus, Naja haje, N. melanoleuca, N. nigricollis*	March 2015 – August 2016
Snake Venom Antiserum Polyvalent (equine)	VACSERA	Egypt	*Bitis arietans, B. gabonica, Cerastescerastes, C. vipera, Echis carinatus, E. coloratus, Macrovipera lebetina, M. palestinae, Naja haje, N. melanoleuca, N. mossambica, N. nigricollis, N. oxiana, Pseudocerastes persicus, Vipera ammodytes, Vipera xanthina, Walterinnesia aegyptia*	January 2017 – August 2018
EchiTabPlus	ICP	Costa Rica	*Bitis arietans, Echis ocellatus, Naja nigricollis*	September 2018 – present
SAIMR Polyvalent	SAVP	South Africa	*Bitis arietans, B. gabonica, Dendroaspis angusticeps, D. jamesoni, D. polylepis, Hemachatus haemachatus, Naja annulifera, N. melanoleuca, N. mossambica, N. nivea*	September 2018 – present
