# Supplementary material for: Treatment outcomes among snakebite patients in north-west Ethiopia—A retrospective analysis
Source: PLoS Negl Trop Dis. 2022 Feb 9;16(2):e0010148. doi: 10.1371/journal.pntd.0010148 (PMC8863263; doi:10.1371/journal.pntd.0010148)
Supplement: S2 Table — (PDF) [file pntd.0010148.s002.pdf]

Supplement 4

Odds of adverse outcome in snakebite patients treated with different antivenoms at Abdurafi field hospital between 2015 and 2019.

		Adverse outcome (%)	Odds ratio (95% CI)	p-value	Adjusted odds ratio*** (95% CI)	p-value
Antivenom						
Fav-Afrique		10/148* (6.8%)	1.00 (reference)			
VacSera		42/164 (25.6%)	4.72 (2.27 - 9.80)	<0.001	6.16 (2.64 - 14.4)	<0.001
EchiTAb-PLUS-ICP		15/155** (9.7%)	1.47 (0.64 - 3.38)	0.367	2.99 (1.12 - 8.00)	0.029
Age (per year)			1.00 (0.97 - 1.02)	0.818	0.99 (0.97 - 1.02)	0.617
Gender	Male	62/414 (15.0%)	1.00 (reference)		1.00 (reference)	0.373
	Female	5/52 (9.6%)	0.60 (0.23 - 1.58)	0.304	0.62 (0.22 - 1.77)	
Time from bite to admission (per day)			1.01 (1.00 - 1.02)	0.159	1.01 (1.00 - 1.02)	0.280
Signs of severe envenomation		37/402 (9.2%)	1.00 (reference)		1.00 (reference)	
		30/64 (46.9%)	8.70 (4.80 - 15.8)	<0.001	8.84 (4.38 - 17.8)	<0.001
Body part bitten	Foot	45/291 (15.5%)	1.00 (reference)	0.911	1.00 (reference)	0.859
	Leg	3/30 (10.0%)	0.61 (0.18 - 2.09)		1.07 (0.27 - 4.21)	
	Hand	16/123 (13.0%)	0.82 (0.44 - 1.51)		1.09 (0.54 - 2.21)	
	Arm	1/7 (14.3%)	0.91 (0.11 - 7.75)		3.05 (0.27 - 33.9)	
	Other	2/15 (13.3%)	0.84 (0.18 - 3.85)		2.12 (0.42 - 10.7)	
Heart rate (per bpm)			1.01 (1.00 - 1.03)	0.044	1.01 (1.00 - 1.03)	0.162
Temperature on admission (per °C)			0.99 (0.85 - 1.16)	0.943	1.01 (0.84 - 1.22)	0.922

* excluding one patient who defaulted treatment (discharged against medical advice)

** excluding one patient who had missing data for heart rate

*** adjusted for age, sex, and time from bite until admission (*a priori*) and signs of severe envenomation (associated with outcome and antivenom in univariate analyses), body part bitten (associated with type of antivenom in univariate analysis), heart rate and temperature on admission (associated with outcome and antivenom in non-parametric tests)
