# Supplementary material for: Treatment outcomes among snakebite patients in north-west Ethiopia—A retrospective analysis
Source: PLoS Negl Trop Dis. 2022 Feb 9;16(2):e0010148. doi: 10.1371/journal.pntd.0010148 (PMC8863263; doi:10.1371/journal.pntd.0010148)
Supplement: S3 Table — (PDF) [file pntd.0010148.s003.pdf]

Supplement 5

Odds of treatment reaction and death in snakebite patients treated with different antivenoms at Abdurafi field hospital between 2015 and 2019.

	n	Outcome (%)	Odds ratio (95% CI)	p-value	Adjusted odds ratio** (95% CI)	p-value
Antivenom		Treatment reaction				
Fav-Afrique	148*	2 (1.4%)	1.00 (reference)			
VacSera	164	30 (18.3%)	16.3 (3.83 - 69.7)	<0.001	16.2 (3.79 - 69.2)	<0.001
EchiTAb-PLUS-ICP	156	11 (7.1%)	5.54 (1.21 - 25.4)	0.028	5.78 (1.25 - 26.7)	0.024
Age (per year)			0.98 (0.95 - 1.01)	0.220	0.98 (0.95 - 1.01)	0.241
Gender (female v male)			0.79 (0.27 - 2.30)	0.661	0.77 (0.25 - 2.31)	0.638
Time from bite to admission (per day)			0.99 (0.97 - 1.01)	0.175	0.99 (0.97 - 1.01)	0.265
Antivenom		Death				
Fav-Afrique	148*	2 (1.4%)	1.00 (reference)			
VacSera	164	8 (4.9%)	3.74 (0.78 - 17.9)	0.097	4.56 (0.90 - 23.0)	0.066
EchiTAb-PLUS-ICP	156	3 (1.9%)	1.43 (0.24 - 8.69)	0.697	1.16 (0.18 - 7.34)	0.871
Age (per year)			1.06 (1.01 - 1.10)	0.009	1.06 (1.02 - 1.11)	0.008
Gender (female v male)			0.65 (0.08 - 5.07)	0.677	0.64 (0.08 - 5.22)	0.677
Time from bite to admission (per day)			1.01 (1.00 - 1.03)	0.097	1.02 (1.00 - 1.03)	0.060

* excluding one patient who defaulted treatment (discharged against medical advice)

** adjusted for all variables in table
