# Supplementary material for: Treatment outcomes among snakebite patients in north-west Ethiopia—A retrospective analysis
Source: PLoS Negl Trop Dis. 2022 Feb 9;16(2):e0010148. doi: 10.1371/journal.pntd.0010148 (PMC8863263; doi:10.1371/journal.pntd.0010148)
Supplement: S1 Form — (PDF) [file pntd.0010148.s004.pdf]

MSF Snakebite Patient Form

Snakebite ID number _ _ _ _ MSF file number _ _ _ _ _ _ Age _____ (Y/M)

Surname ______________________ First name _________________ Sex M ◯ F ◯

Snakebite time Date _ _ / _ _ / _ _ Time _ _ : _ _

Local practitioner consultation Date _ _ / _ _ / _ _ Time _ _ : _ _

MSF Consultation/Admission Date _ _ / _ _ /_ _ Time _ _ : _ _

Part of body bitten : foot ◯, leg ◯, hand ◯, arm ◯, trunk ◯, face ◯; left ◯ right ◯

Location of bite (village) : ___________indoors ◯, compound ◯; field ◯; road ◯; tree ◯; river ◯

Context: sleeping ◯, playing ◯, walking◯, working ◯, cutting grass ◯, toilet ◯;

Snake description: size small <1m ◯ large >1m ◯, black ◯ brown ◯ green ◯ diamond pattern ◯

Suspected species : ____________(check photo album) ◯ snake not seen

Initial Presentation : HR _ _ _ BP _ _ / _ _ T° _ _,_ CRT _ _ sec

1. **Cytotoxic:** Local swelling (not progressive) ◯ Progressive swelling (>1joint) ◯; necrosis ◯
2. **Haemotoxic:** Spontaneous bleeding ◯ 20 min WBCT Non-clotting ◯ clotting ◯ not done ◯
3. **Neurotoxic:** Ptosis (droopy eyes) ◯, diplopia◯, blurred vision◯, difficulty talking◯, swallowing◯, breathing◯, confusion ◯ needs oxygen ◯ (SaO2 _ _ _%)
4. **For all: BP __/__ ;** Shock Yes ◯ No ◯ // Conscious ◯ Unconscious ◯ (no response to pain)

Antivenom: **Not needed ◯, Fav-Afrique ◯, EchiTabPlus ◯, SAIMR/SAVP◯, other◯**

1st treatment # Vials used: ____ date _ _/ _ _ time_ _:_ _

If need: 2nd treatment # Vials used: ___ date _ _/ _ _ time_ _:_ _

3rd ◯ date _ _/ _ _ time_ _:_ _ More ? (explain) ________________________________

Time of improvement start: date _ _/ _ _ time_ _:_ _ Time of cure: date _ _/ _ _time_ _:_ _

Adverse reactions: skin itchy rash (urticarial/hives) ◯; abdominal pain◯; vomiting ◯; difficulty breathing-stridor ◯ asthma-wheezing ◯ hypotension (BP __/__) ◯ shock ; High Temp >38° ◯ ;

Time of 1st adverse reaction Date _ _ / _ _ / _ _ Time _ _ : _ _

Delayed reaction (joint pain & fever) Date _ _ / _ _ / _ _ Time _ _ : _ _

Other treatments: IV Ringer infusion ◯ adrenalin ◯ antihistaminic ◯ other ◯ ________

Complications: Severe necrosis+disability ◯; Amputation ◯; Anxiety-PTSD ◯; other ______

MSF Discharge: Date _ _ / _ _ / _ _ Time _ _ : _ _

Final diagnostic: **1. cured ◯ 2. cured with sequelae ◯ 3. death ◯ 4. defaulted ◯**
