# Supplementary material for: Treatment outcomes among snakebite patients in north-west Ethiopia—A retrospective analysis
Source: PLoS Negl Trop Dis. 2022 Feb 9;16(2):e0010148. doi: 10.1371/journal.pntd.0010148 (PMC8863263; doi:10.1371/journal.pntd.0010148)
Supplement: Attachment — Submitted filename: 20210924 Reponse Letter Snakebite research Ethiopia.pdf [file pntd.0010148.s006.pdf]

Response to reviewers comments

Epidemiology, treatment outcomes and antivenom effectiveness in snakebite patients in north-west Ethiopia – a retrospective analysis.

Inge Steegemans, Kassaye Sisay, Ernest Nshimiyimana, Gashew Gebrewold, Turid Piening, Endale Menberu Tessema, Birhanu Sahelie, Gabriel Alcoba, Fikre Seife, Dirk Essink, Emiliano Lucero, Koert Ritmeijer.

Unknown reviewer

Introduction:

1. Line 102, "Moreover, little data is available on the effectiveness and safety of different antivenoms"
   Please clarify, is this statement referring to Africa?

Response:
Yes, this statement is referring to Africa, and this has been added in the text. We included the following reference [17] in order to clarify this:
Potet J, Smith J, McIver L (2019) Reviewing evidence of the clinical effectiveness of commercially available antivenoms in sub-Saharan Africa identifies the need for a multi-centre, multiantivenom clinical trial. PLoS Negl Trop Dis 13(6): e0007551. https://doi.org/10.1371/journal.pntd.0007551

2. Line 118, "First, the Fav-Afrique antivenom (Sanofi Pasteur, France) was used, which is a polyvalent antivenom and is effective against most bites of medically important snakes in sub-Saharan Africa"
   Mention the snake species the Fav-Afrique antivenom is developed against.

Response:
We added the snake species Fav-Afrique is developed against (line 122-123). Details on the snake species and the different antivenoms have been developed for are presented in Supplement Appendix 1.

Methods:

3. Since the different antivenoms were used at different periods, one will wonder if other supportive care the patients also received at these different periods were also similar across the groups. For example, in the result section you mentioned that the time delay between presentation to hospital and antivenom administration was 1.5 hours for Fav-Afrique, while For VacSera it was 6 hours and for EchiTAb-PLUS-ICP it was 9 hours. This may suggest difference in standard of care at these different periods. Therefore, there is need for a statement on how similar the supportive care the different patient groups received, since these may also affect the outcome. If the data is available it will be useful to reflect it in table 1 showing the baseline characteristics of the different groups.

Response:
Over the years there were no major changes made in first aid care for snakebite patients. However, over time patients did present earlier which is most likely due to the increased community awareness, partly due to health promotion activities. When patients present to the clinic early after a snakebite, the healthcare workers have more time to observe the patient and to decide whether or not antivenom

administration is needed. There is no sufficient data available on the supportive patient care, however, we added a paragraph discussing these aspects in the discussion (line 366-378).

Results:

4. Line 243, "the mean time between admission to the clinic and the administration of antivenom for Fav-Afrique was 1.5 hours. For VacSera this time was 6 hours and for EchiTAb-PLUS-ICP this was almost 9 hours." The time difference if statistically significant can affect the groups' outcome as a confounder. Firstly, you need to show if the difference is statistically significant and if so how the confounder effect was addressed.

Response:
We assessed whether the variable ''the time between admission to the clinic and the administration of antivenom'' could be a possible confounder for the study using a Kruskal Wallis test. The time difference between the admission to the clinic and the administration of antivenom among the three groups of antivenom was not statistically significant. We added the results of the test of significance to table 2 to show which variables could have affected the groups' outcomes.

5. Line 352, "The main types of complications that occurred during the treatment were severe pain, extensive internal or external bleeding, severe swelling, and fever/infection of the bite site. However, in most cases, these complications can be attributed to the venom-induced syndrome."
   With exception of fever/infections these are rather manifestations of envenomation rather than complications.

Response:
This is a fair point. In order to be more precise, we changed the name 'complication(s)' into 'severe envenomation' when talking about signs that are manifestations of the envenomation, such as severe pain, swelling and bleeding. When mentioning signs such as fever/infection or necrosis the term 'complication' is used. In order to improve the quality and the readability of the manuscript we have decided to leave out the outcome variable ''signs of severe envenoming'' since this is not directly an indicator of the effectiveness of the different antivenoms.

Reviewer #1

Methods
(No response)

Results
(No response)

Conclusion
(No response)

Editorial and dada presentation modifications
(No response)

Summary and general comments

Thank you for the opportunity to review “Epidemiology, treatment outcomes and antivenom effectiveness in snakebite patients in north-west Ethiopia – a retrospective analysis”. This is an important topic in an understudied area, and I think this paper will make a good addition to the literature. Although I think it’s quite interesting, I do have some problems with the statistics and feel revisiting this section with different tools would make these results more useful for clinicians and policy makers.

Major comments:

1. Using Pearsons correlation is an unusual test to compare independent variables and your outcome of treatment (poor/ recovered). Pearsons works for normally distributed variables, and isn’t a great choice for binary outcomes; are you sure the variables meet these conditions? The correlation coefficient you present in Table 6 is hard to interpret and thus not very useful. Because you have a limited number of outcomes, you don’t have much statistical power for multivariable modeling, but I would recommend instead presenting the results from a logistic regression to show the associations between patient characteristics and outcome (i.e. one variable at a time). Presenting this as an odds ratio (or prevalence ratio, which is preferred though perhaps less common) with the 95% CI (and perhaps p value if you wish) will give the reader more informative data on the magnitude, precision, and significance of the association between each set of variables. You may wish to consider a
   limited multivariable analysis (e.g., one predictor variable, controlling for antivenom type and maybe time/year?) but you have a limited number of outcomes, so this may not be feasible. \
   Regarding using ANOVA and your post-hoc tests, the post hoc tests use a selective component of the shared variance for all the groups taken from the overall F statistic. That said, the sample sizes in the groups are different, and Tukey-HSD test is for samples of the same size. You may wish to consult with your statistician, I think you probably should have used a Tukey-Kramer test.

Response:
Thank you for these important comments. We have changed the ANOVA analysis to presentation of odds ratios, 95% CI and p-value when comparing the different antivenoms for the outcome of treatment and the development of adverse reactions to the antivenom.

For table 6 (now table 5), we have chosen to use a Spearman correlation test instead of Pearson due to the conditions for a Pearson test. Unfortunately, we were not able to perform a regression with our data, due to the small sizes of different groups.

2. Line 237: Can you say anything about the significance of differences across groups of patients? It looks like the FAV-Afrique patients took longer to come in to the hospital. Is this a ‘statistically significant’ difference that you may wish to control for, or are these means and medians not meaningfully different? Since you have so few outcomes as to make multivariable analysis quite limited, I would urge you to compare your populations (i.e., show a p value across groups) so the reader can be aware of where differences lie.

Response:
Thank you for this suggestion, this is indeed very useful. We have added p-values across the groups in table 2 in order to present whether there are significant differences between the groups, or not.

3. Table 3: I'd recommend showing the number and percent of persons with the outcome of interest, rather than the "mean" or proportion as is currently shown (i.e., first column shows total persons who received treatment, second column shows total number with positive outcome, third column shows the percentage XX.X% of persons with positive outcome). Same recommendation applies to Tables 4 and 5.

Response:
We took table 3, 4 and 5 out and replaced them with revised tables 3 and 4, showing the comparison of antivenoms with %, OR, 95%-CI and p-values.

Minor comments:

4. When in 2015 did you begin providing antivenom (i.e., what month did you start administering Fav-Afrique)? It would be appreciated if you could make more precise your duration-of-use estimates in line 115-116. Could you also specify in this section of the Introduction: what was the standard of care prior to MSF's arrival?

Response:
Fav-Afrique antivenom was introduced in Ethiopia by MSF in March 2015. Standard care before antivenom was available consisted of first aid and supportive care and management. Also referral to a secondary or tertiary hospital was provided to snakebite patient who were in need of more intensive treatment or surgery, due to severe envenoming. This information is added to the introduction (line 114-117).

5. Line 162: Please describe a bit more about those who did not receive antivenom. Did all in this category demonstrate symptoms (Table 1 suggests this is the case)?

Response:
Patients in the 'no-antivenom'-group all had symptoms, but only in a mild form compared to patients who were in need of antivenom, i.e. only local swelling, no signs of active bleeding. Symptoms diminished within a maximum of a few days. This information is added to the description of table 1 (line 245-247).

6. Is there anyone in this category who should have received antivenom but did not for whatever reason?

Response:
There was a period during the research period, September 2016 until January 2017, that there was no antivenom available as the last batch of Fav-Afrique expired in September 2016 and there was no alternative antivenom available yet until January 2017. Less patients admitted to the clinic compared to other periods, possibly because word had spread that there was no antivenom available. However, there were still a few patients (n = 11) who had admitted to the clinic who were not able to receive antivenom treatment.

7. What about patients who come who do not exhibit symptoms (e.g., they were bitten, but the envenomation was mild/nonexistent) – were there any patients who really didn't need much help at all, but were just worried?

Response:
All snakebite patients presenting at the facility were admitted for observation and received analgesics and mental health support, even in case of what eventually was diagnosed as a dry bite. So yes, there were patients who admitted to the clinic who didn't need much help.

8. Does this category also draw on patients admitted during the gap after Fav-Afrique expired and no treatment was available? I would assume this would then bias the type of patient included as a comparison (they'd be more likely to be in high need of antivenom even though they could not get it)

Response:
Yes, as mentioned above at question number 6, there were 11 patients who admitted to the clinic during the antivenom-gap after Fav-Afrique expired. In order to exclude possible bias, we excluded the patients admitted between September 1st 2016 and December 31th 2016 from the analysis. We added this to the exclusion criteria (line 171-174).

9. You mention the SAIMR polyvalent antivenom in line 127, but this is not shown as one of the antivenoms used in lines 115-116. Please clarify, or add this antivenom. (edit: I see you mention this in line 166. I would recommend that you clarify that too few patients were treated with SAIMR to be included in the analysis in lines 115-6)

Response:
This is added in line 119, and explained in the methods that only one patient was treated with SAIMR during the research period, and therefore SAIMR is not included in the analysis.

10. Table 2: what is "defaulted"? Is that lost to follow up/unknown? Could you edit this last variable to match your outcome as defined in the methods (i.e., "cured" vs. "poor" – but including death as one of the "poor" outcomes as that's informative)

Response:
The term ''defaulted'' is indeed a bit vague. Patients who were defaulted are basically patients who were discharged against medical advice. We changed ''defaulted'' into ''discharge against advice'' in table 2 to make this more clear.

11. Minor grammatical issues throughout, e.g., Line 36+: "Since 2015 Médecins Sans Frontières is treating" could be changed to "Since 2015 Médecins Sans Frontières has been treating";

Response:
Correct, thank you.

12. Line 70 (and same comment for line 71): “This study shows that patients treated with VacSera antivenom have a higher chance on a poor outcome” should be “have a higher chance of a poor outcome”

Response:
Correct, thank you.

13. Lines 260 and 267 “Tuckey” should be “Tukey”. These are mostly minor, as I did not see any grammatical issues that interfered with my ability to interpret your message.

Response:
Correct, thank you. We took out the ANOVA comparisons so the term ‘’Tukey’’ as well.

Reviewer #2

Methods
The objectives of the study were clearly stated. The study was appropriately designed to address the stated objectives and the study population was clearly described and appropriate to to test the hypothesis. Correct statistical analysis were used to arrived at the conclusion. All the ethical and regulatory requirement were met.

Results
The analysis presented matched the analysis plan, and the results were clearly and completely presented. The tables and figures were of sufficient quality and clarity.

Conclusion
The conclusions were supported by the data presented. The manuscript and the data presented are elaborate and help in advancing the understanding of the subject matter. The public health relevance has been addressed.

Editorial and dada presentation modifications
The authors looked at the effectiveness of 3 different antivenoms that were used at different periods on outcome of snakebite envenoming. Differences in time between admission and antivenom administration were reported across the three groups, however, it was not shown if this difference is statistically significant. This is relevant because time delay between hospital admission and antivenom administration negatively affect outcome. The authors need to address this and provide information on how the confounder effect was addressed.

Response:
We assessed whether the variable ‘’the time between admission to the clinic and the administration of antivenom’’ could be a possible confounder for the study using a Kruskal Wallis test. The time difference between the admission to the clinic and the administration of antivenom among the three groups of antivenom was not statistically significant. We added the results of the test of significance to table 2 to show which variables could have affected the groups’ outcomes.

Summary and general comments

Data on the effectiveness and safety of the many antivenoms available in Africa is sparse and this has a huge implication on the management of snakebite envenoming in the continent. The manuscript therefore, provides a vital information on this subject matter. However, the retrospective nature of the study is a weakness as some vital information were not captured. Nevertheless, the authors must be recommended by providing an insight on this important issue and pave a way for further prospective study.

Response: We would like to thank the reviewer for the kind words.

Reviewer #3

Methods

The objectives and study design are articulated and appropriate statistics used for analysis. Ethics approval in place.
That said the following needs attention:

1. The data collection tool needs to be provided - perhaps as a supplementary table

Response:
We added the data collection tool, the snakebite patient form, as a supplement to the manuscript (Supplement Appendix 2).

2. Patient inclusion/exclusion criteria need to be carefully described

Response:
The inclusion and exclusion criteria of this study are described in ''Sampling procedure'' in the methods-section. Every patient who was admitted to the clinic between January 2015 and December 2019 and received antivenom-treatment was included. For the patient group who did not receive antivenom treatment, Grays' systematic sampling strategy was used where every third chronological patient was included in the study.
During the research period, there was 1 patient who received SAIMR antivenom. Since this was only 1 patient, this patient was excluded from the analysis. In addition, we excluded all patients who were admitted to the clinic between September 1st 2016 and December 31th 2016 to exclude any bias due to the antivenom supply gap. Please see also the response on question number 8 of reviewer 1.

3. The SAVP product - SAIMR poly is described in the introduction as being used by MSF in this hospital but is not included in the analysis - an explanation for this exlucsion is needed

Response:
This is explained in the methods section (line 175 - 177). Because there was only one patient treated with SAIMR antivenom this patient was excluded from the analysis. The SAIMR antivenom was reserved for patient with a neurotoxic envenoming, or in critical cases of severe cytotoxic envenoming. These cases have been very rare.

4. A table describing the antivenoms used is needed (akin to that used by the Potet et al review of antivenoms) particularly with regards the venoms used for manufacture - as a supplementary table

Response:
Thank you for this suggestion, we added a table similar to the table 2 used by Potet et.al. as a supplement to give more information about the different antivenoms that were used (Supplement Appendix 1).

Results
This is a very important paper that makes several important points about snakebite management in sub-Saharan Africa but it requires careful editing to maximise its impact. The grammar, spelling and phrasing needs attention:

5. the name of the ICP antivenom is EchiTAb-PLUS-ICP - correct this throughout.

Response:
Thank you for pointing this out, we replaced ''EchiTabPlus'' with ''EchiTAb-PLUS-ICP''.

6. antivenoms are often described as 'incorrect' or 'wrong' - please correct this inadequate description throughout and make it understandable to a non-snakebite audience - using a reference to the 'antivenoms' table suggested elsewhere in this review.

Response:
We corrected the term "wrong antivenom" to "unsuitable product, which has not been developed to neutralize venoms of relevant endemic snake species".

I have the following questions regarding data management and results presentation:

7. I'm not a statistician but isn't a probability of 0.000 meaningless? Should it be $p<0.005$?

Response:
True, we changed 0.000 to $p<0.001$.

8. much of the data/analysis is upon 'complications' but this term is inadequately defined - the methods section would benefit from a section defining this, and subsequent analyses.

Response:
Thank you for pointing this out. We changed the term "complications of the bite" into "signs of severe envenoming" when talking about signs that are manifestations of the envenomation, such as severe pain, swelling and bleeding. When mentioning signs such as fever/infection or necrosis the term 'complication' is used. In order to improve the quality and the readability of the manuscript we have decided to leave out the outcome variable ''signs of severe envenoming'' since this is not directly an indicator of the effectiveness of the different antivenoms (also see point 5 from ''unknown reviewer''.

9. Table 7 focuses upon 'complications' but includes only 1 distinct analysis criteria from those in Table 6, which focuses upon Rx outcomes - why is this duplication needed?
10. And why are the figures for many of these identical criteria different in Tables 6 and 7?
11. In fact- please review both these Tables VERY carefully - because Table 6 (Rx outcome analysis) has a 'complications' analysis criteria but not a 'poor outcome' criteria, whereas Table 7 (Rx complications analysis) does NOT have a 'complications' analysis criteria. Please fix this major confusion and remove duplicated data (that are numerically inconsistent between the tables).

Response:
We understand the confusion. In order to avoid this we chose to take out table 7 as the analysis in table 6 (outcome of treatment) is more important. You are correct when you state that the criteria is almost identical in both table 6 and 7. The difference in the numbers in the tables is due to the fact that in table 6 the dependent variable is whether the patient experienced a poor outcome of the total treatment, and in table 7 the dependent variable is whether a patient experienced complications during the treatment. This leads to different figures. However, looking back, we consider the analysis in table 7 as not quite relevant to this study. Hence, we have chosen to take it out and only display results that really say something.

12. The bar charts for Figure 1 should be in colour to enable greater clarity of the change in annual admission rates - this is an important part of the study's finding and deserves greater clarity

Response:
The Intention is indeed that Figure 1 will be published in color.

<u>Conclusion</u>

13. The conclusions are supported by the data analysis, with the exception that assigning snake species to hospital admissions (Table 1) is not supported by identification methods. If the authors want to include this (which I suggest they don't because it weakens the study's accuracy - see very speculative assumptions Abstract line 48/9), this entry should read 'Suspected snake species responsible for admission'). In this context - please be more careful using snake species names - the saw-scaled vipers in Egypt are either Echis coloratus or E. pyramidum - only the later is present in Ethiopia. It is correctly termed the North East African saw-scaled viper.

Response:
Thank you for pointing this out. In the abstract, sentence 48-49, we replaced ''caused by'' with "attributed to", to clarify that we are not 100% sure of this. In the methods section we added an explanation on how the health workers try to identify the snake species responsible for the bite: "Suspected snake species was assessed by patient description of the snake if possible, using a snake atlas with pictures and sizes. Snakes caught by a snake catcher of the Ethiopian Public Health Institute on the farms in Abdurafi area in 2017 identified *Echis pyramidum* and *Bitis arietans* as the main venomous snakes in the area." In table 1 we changed ''snake species'' to 'suspected snake species''.

14. Antivenom affordability is correctly described as a major issue in snakebite management - but is not analysed here. Can this data and analysis be included here, especially in terms of patient admission, outcome and adverse effects?

Response:
Although antivenom affordability is a major factor in patient access to appropriate snakebite care, affordability was not an issue in this study, because antivenoms treatment and all supporting care and hospitalization were provided free of charge in the MSF hospital. We added this information to the discussion of the study (line 396 – 398).

15. The higher rate of complications when using FavAfrique (line 349) deserves a much more comprehensive discussion - and was lower than Vacsera (Table 7)

Response:
The higher rate of severe envenomation when using Fav-Afrique is most likely associated with delayed health seeking due to lower community awareness of the free treatment and services provided by the MSF clinic. We have added this explanation to the discussion (line 327 – 329).

16. Cite ref 35 for high adverse effects of the Vacsera product.

Response:
Thank you.

17. Paragraph starting line 304. The NE Nigeria snakebite-research team led by Prof Habib has also noted that admission rates increase when availability of antivenom becomes known in communities - I suggest you seriously consider citing ref 39 here (or other papers by this research team) to emphasise that this aspect of health seeking behaviour is evident in different settings. Its an important point to improve snakebite management.

Response:
Thank you for pointing this out. We added the research by Prof Habib as a reference to this part of the discussion.

I have made several critical comments but only in an attempt to improve the impact and uptake of this very important paper that delivers many important points - that hopefully will receive attention and action from appropriate national, regional and international agencies.

Editorial and dada presentation modifications
Please review the grammar, spelling and phrasing throughout.

In addition to the many points already made:

18. Line 131 - SAIMR is manufactured with, and effective against, venoms from many Bitis vipers, including puff adders

Response:
Correct. We added the following sentence to line 133-135 in order to make this clear: “SAIMR is an antivenom used in case of neurotoxic envenoming by mambas (*Dendroaspis spp.*) and spitting and non-spitting cobras (*Naja spp.*), **and in critical cases of severe cytotoxic envenomation by *Bitis* vipers.** “ Supplement Appendix 1 provides the details on the antivenoms.

19. I really dislike the inclusion of several non-authorative, non peer-reviewed, internet references in the list of citations. This seems lazy referencing to me - especially refs 10, 15. There exist several other, peer-reviewed papers that make the points that the authors seek to support their themes.

Response:
Thank you for this feedback. We took out reference 10, 14 and 15 and we replaced them with the following:
Warrell DA, Unscrupulous marketing of snake bite antivenoms in Africa and Papua New Guinea: choosing the right product—'What's in a name?', Transactions of the Royal Society of Tropical Medicine and Hygiene, Volume 102, Issue 5, 2008,

Brown NI (2012) Consequences of Neglect: Analysis of the Sub-Saharan African Snake Antivenom Market and the Global Context. PLoS Negl Trop Dis 6(6): e1670.

We also edited the reference list and took out the ''[Internet]'' and ''[cited at…]''.

<u>Summary and general comments</u>
As stated before, I think this is an important paper to our filed, whose findings need to be widely disseminated and acted upon.

I have made several critical comments but only in an attempt to improve the impact and uptake of this very important paper that delivers many important points - that hopefully will receive attention and action from appropriate national, regional and international agencies.

Response: Thank you all for putting your effort in reviewing this paper and providing these useful comments.
