# Supplementary material for: Treatment outcomes among snakebite patients in north-west Ethiopia—A retrospective analysis
Source: PLoS Negl Trop Dis. 2022 Feb 9;16(2):e0010148. doi: 10.1371/journal.pntd.0010148 (PMC8863263; doi:10.1371/journal.pntd.0010148)
Supplement: Attachment — Submitted filename: 20211213 Response letter snakebite research Ethiopia.pdf [file pntd.0010148.s007.pdf]

Responses to Reviewers' Comments

Treatment outcomes among snakebite patients in north-west Ethiopia – a retrospective analysis.

Inge Steegemans, Kassaye Sisay, Ernest Nshimiyimana, Gashew Gebrewold, Turid Piening, Endale Menberu Tessema, Birhanu Sahelie, Gabriel Alcoba, Fikre Seife, Dirk Essink, Simon Collin, Emiliano Lucero, Koert Ritmeijer.

Reviewer #4

1. It is stated this study provides data on incidence - I don't think this is correct as there is no denominator data.

Response:
'Incidence' changed to 'frequency' in several places where former term was used incorrectly.

2. As this is a non-randomised study that aims to comapre safety and effectiveness of 3 antivenoms, much more needs to be done to adjust for baseline differences/confounders. The statistical analysis is basic and there is no adjustment for baseline differences between the groups.

Response:
We have amended the title to clarify that this non-randomised study does not determine relative effectiveness and instead is an analysis of adverse outcomes among patients who did and did not receive antivenom. We have conducted a completely fresh analysis of adverse outcomes as a binary outcome in a logistic regression model adjusted for age, sex, signs of severe envenomation, body part bitten, heart rate and temperature on admission, and time from bite until admission.

3. There is insufficient information on how the outcomes were allocated as 'cured' vs 'poor outcome.' Was this a single person or two independent people? Was this person blinded to the treatment outcome? More information on how 'cured' was defined is needed: in particular, whether this was objective or open to interpretation.

Response:
Throughout the manuscript, the term 'cured' was amended to 'uncomplicated outcome' and 'poor outcome' to 'adverse outcome'. We have clarified in our methods that patients were classified into this binary outcome using routinely collected data as described in the 'Data collection and analysis' section. This was done by one person as part of data preparation.

4. As highlighted above, major changes are required in the analysis. Table 1 should list all confounders that may account for differences in treatment outcome: particularly any differences in severity of envenoming at baseline. Degree of swelling, proprortion with systemic bleeding, and proportion with abnormal 20WBCT at baseline are important. If there are differences betweent the groups at baseline then either comparisons of the antivenoms should not be done, or adjustment for the baseline differences between the groups needs to be undertaken. Consider matching, or more advanced statistical techniques for adjusting for baseline differences. As this is a non-randomised study, it is highly vulnerable to bias and this needs to be made up for with carefully considered analysis. I would suggest seeking further support from an experienced statistician.

Response:
We have simplified our 'adverse outcome' variable to include adverse reactions and conducted a logistic regression with type of antivenom as primary exposure, presenting crude odds ratios and

odds ratios adjusted for age, sex, and time from bite until admission (included as *a priori* confounders) and signs of severe envenomation, body part bitten, heart rate and temperature on admission (associated with outcome and type of antivenom, not considered to be on a causal pathway, and with no missing data).

5. As this is retrospective, there must have been missing and innacurate data. This needs to be detailed.

Response:
Missing data were indicated in Table 1 but not in Table 2. This information has been added to Table 2. Table 3 is based on complete data (none missing). We have commented in the text that we were unable to assess whether to adjust for time from admission to administration of antivenom because these data were missing for 167/468 patients.

6. I don't think Spearman's Rho is the best analysis for Table 5. Multivariate logistic regression analysis would be preferrable. Reduce the number of predictor variables if there are issues with small numbers. Have missing data prevented undertaking multivariate logistic regression analysis?

Response:
All correlation analysis has been removed. The main analysis now is multivariable logistic regression as suggested. We simplified our 'adverse outcome' variable to include adverse reactions and conducted a logistic regression with type of antivenom as primary exposure, presenting crude odds ratios and odds ratios adjusted for age, sex, and time from bite until admission (included as *a priori* confounders) and signs of severe envenomation, body part bitten, heart rate and temperature on admission (associated with outcome and type of antivenom, not considered to be on a causal pathway, and with no missing data).

7. Suggest revising cited articles in first paragraph: Reference 1 – avoid referencing opinion type articles for epidemiology. Consider Kasturiratne 2008. Reference 3 seems incorrect for epidemiological statement?

Response:
Thank you for the suggestion. We replaced the opinion type article with the suggestion you made: Kasturiratne, 2008. The reference concerning the epidemiological statement was falsly placed and is replaced by an article by Potet, 2019.

8. Overall this is an important and well written paper. The dataset is of a reasonable sample size and seems to be of good quality. Unfortunately I think the study cannot be published without making fairly major changes to the analysis. Non-randomsied studies such as this are highly vulnerable to bias and careful analysis is needed to overcome this. This could change the main finding of the paper. Comparing crude proportions with 'cure' vs 'poor outcome' can be misleading. Once this is done, the paper would be of high quality and would certainly be suitable for publications in PLOS NTD.

Response:
We thank the reviewer for their encouragement and insightful comments. As per responses above, we have conducted a complete re-analysis using logistic regression. Although this allowed us to adjust for confounders, we have acknowledged in our discussion that unmeasured and residual confounding will still be a limitation of this analysis and that our analytical dataset represented a modest proportion of all patients treated during the study period.
