# Supplementary material for: Treatment outcomes among snakebite patients in north-west Ethiopia—A retrospective analysis
Source: PLoS Negl Trop Dis. 2022 Feb 9;16(2):e0010148. doi: 10.1371/journal.pntd.0010148 (PMC8863263; doi:10.1371/journal.pntd.0010148)
Supplement: Attachment — Submitted filename: Reviewers comment plos NTD.docx [file pntd.0010148.s005.docx]

Introduction:

1. Line 102, “Moreover, little data is available on the effectiveness and safety of different antivenoms”

Please clarify, is this statement referring to Africa?

2. Line 118, “First, the Fav-Afrique antivenom (Sanofi Pasteur, France) was used, which is a polyvalent antivenom and is effective against most bites of medically important snakes in sub-Saharan Africa”

Mention the snake species the Fav-Afrique antivenom is developed against.

Methodology:

1. Since the different antivenoms were used at different periods, one will wonder if other supportive care the patients also received at these different periods were also similar across the groups. For example, in the result section you mentioned that the time delay between presentation to hospital and antivenom administration was 1.5 hours for Fav-Afrique, while For VacSera it was 6 hours and for EchiTabPlus it was 9 hours. This may suggest difference in standard of care at these different periods. Therefore, there is need for a statement on how similar the supportive care the different patient groups received, since these may also affect the outcome. If the data is available it will be useful to reflect it in table 1 showing the baseline characteristics of the different groups.

Results:

1. Line 243, “the mean time between admission to the clinic and the administration of antivenom for Fav-Afrique was 1.5 hours. For VacSera this time was 6 hours and for EchiTabPlus this was almost 9 hours.”

The time difference if statistically significant can affect the groups’ outcome as a confounder. Firstly, you need to show if the difference is statistically significant and if so how the confounder effect was addressed.

2. Line 352, “The main types of complications that occurred during the treatment were severe pain, extensive internal or external bleeding, severe swelling, and fever/infection of the bite site. However, in most cases, these complications can be attributed to the venom-induced syndrome.”

With exception of fever/infections these are rather manifestations of envenomation rather than

complications.
